# Implementing a New Algorithm for Reinterpretation of Ambiguous Variants in Genetic Dilated Cardiomyopathy

**DOI:** 10.3390/ijms25073807

**Published:** 2024-03-29

**Authors:** Alexandra Pérez-Serra, Rocío Toro, Estefanía Martinez-Barrios, Anna Iglesias, Anna Fernandez-Falgueras, Mireia Alcalde, Mónica Coll, Marta Puigmulé, Bernat del Olmo, Ferran Picó, Laura Lopez, Elena Arbelo, Sergi Cesar, Coloma Tiron de Llano, Alipio Mangas, Josep Brugada, Georgia Sarquella-Brugada, Ramon Brugada, Oscar Campuzano

**Affiliations:** 1 Cardiovascular Genetics Center, Institut d’Investigació Biomèdica de Girona (IDIBGI-CERCA), Parc Hospitalari Martí i Julià, Edifici M2, 17190 Salt, Spain; aperez@idibgi.org (A.P.-S.); annai@brugada.org (A.I.); annafernandez.girona.ics@gencat.cat (A.F.-F.); malcalde@gencardio.com (M.A.); monicacoll.girona.ics@gencat.cat (M.C.); mpuigmule.girona.ics@gencat.cat (M.P.); bdelolmo@gencardio.com (B.d.O.); ferran.pico@gencardio.com (F.P.); llopez@gencardio.com (L.L.); 2 Centro de Investigación Biomédica en Red Enfermedades Cardiovasculares (CIBERCV), 28029 Madrid, Spain; elenaarbelo@secardiologia.es (E.A.); josep@brugada.org (J.B.); 3 Medicine Department, School of Medicine, Cadiz University, 11003 Cadiz, Spain; rocio.toro@uca.es (R.T.); alipio.mangas@uca.es (A.M.); 4 Research Unit, Biomedical Research and Innovation Institute of Cadiz (INiBICA), Puerta del Mar University Hospital, 11009 Cadiz, Spain; 5 European Reference Network for Rare, Low Prevalence and Complex Diseases of the Heart (ERN GUARD-Heart), 1105 AZ Amsterdam, The Netherlands; estefania.martinez@sjd.es (E.M.-B.); sergi.cesar@gmail.com (S.C.); georgia@brugada.org (G.S.-B.); 6 Pediatric Arrhythmias, Inherited Cardiac Diseases and Sudden Death Unit, Cardiology Department, Sant Joan de Déu Hospital de Barcelona, 08950 Barcelona, Spain; 7 Arrítmies Pediàtriques, Cardiologia Genètica i Mort Sobtada, Malalties Cardiovasculars en el Desenvolupament, Institut de Recerca Sant Joan de Déu, Esplugues de Llobregat, 08950 Barcelona, Spain; 8 Cardiology Service, Hospital Josep Trueta, University of Girona, 17007 Girona, Spain; ctiron.girona.ics@gencat.cat; 9 Arrhythmias Unit, Hospital Clinic, University of Barcelona-IDIBAPS, 08036 Barcelona, Spain; 10 Internal Medicine Department, Puerta del Mar University Hospital, School of Medicine, University of Cadiz, 11009 Cadiz, Spain; 11 Medical Science Department, School of Medicine, University of Girona, 17003 Girona, Spain

**Keywords:** sudden cardiac death, dilated cardiomyopathy, genetics, interpretation

## Abstract

Dilated cardiomyopathy is a heterogeneous entity that leads to heart failure and malignant arrhythmias. Nearly 50% of cases are inherited; therefore, genetic analysis is crucial to unravel the cause and for the early identification of carriers at risk. A large number of variants remain classified as ambiguous, impeding an actionable clinical translation. Our goal was to perform a comprehensive update of variants previously classified with an ambiguous role, applying a new algorithm of already available tools. In a cohort of 65 cases diagnosed with dilated cardiomyopathy, a total of 125 genetic variants were classified as ambiguous. Our reanalysis resulted in the reclassification of 12% of variants from an unknown to likely benign or likely pathogenic role, due to improved population frequencies. For all the remaining ambiguous variants, we used our algorithm; 60.9% showed a potential but not confirmed deleterious role, and 24.5% showed a potential benign role. Periodically updating the population frequencies is a cheap and fast action, making it possible to clarify the role of ambiguous variants. Here, we perform a comprehensive reanalysis to help to clarify the role of most of ambiguous variants. Our specific algorithms facilitate genetic interpretation in dilated cardiomyopathy.

## 1. Introduction

Dilated cardiomyopathy (DCM) is an entity characterized by left ventricular (LV) or biventricular dilatation in the absence of hypertension, valve disease, or coronary artery disease [1]. The heterogeneous etiology of DCM includes wide-ranging genetic and acquired disorders, leading to heart failure and the main cause of transplantation in young people worldwide.

Nowadays, a genetic origin of DCM (G-DCM) can be found in 20–30% of patients with DCM, increasing to 30–50% in those with a family history, mainly following an autosomal dominant pattern of inheritance [2]. DCM can manifest during adolescence or in young adults despite penetrance being age-dependent, and careful diagnostic work-up should be performed to identify the underlying cause and then consider an etiology-oriented management [1]. Nowadays, several rare alterations in more than 50 genes have been associated with G-DCM, despite comprehensive analysis concluding that many of these genes have limited or disputed evidence of causality [2,3]. Truncating variants in the titin gene (*TTN*) are found in up to 20% of G-DCM cases, being the most frequent cause of DCM in adults, so far. In pediatric populations, G-DCM is usually also associated with rare variants of *TTN*, but aggressive phenotypes have been also associated with deleterious variants in *MYH7*, *TNNT2*, *RBM20*, and *LMNA*. Additional deleterious variants related to G-DCM have also been identified in the following genes, although only a definite/robust association should be taken into account in clinical diagnosis: *ACTC1*, *ACTN2*, *BAG3*, *DES*, *DSP*, *FLNC*, *JPH2*, *LMNA*, *MYH7*, *NEXN*, *PLN*, *RBM20*, *SCN5A*, *TNNC1*, *TNNI3*, *TNNT2*, *TPM1*, and *VCL* [2,4,5].

In 2015, the American College of Medical Genetics and Genomics (ACMG) structured standard terminology for classifying sequence variants that help clinical translation [6]. Identification of an alteration definitively classified as pathogenic or likely pathogenic (P/LP) following ACMG recommendations confirms the genetic origin and helps distinguish G-DCM from other cardiomyopathies or overlapped phenotypes. A recent study has shown an increased risk of developing cardiomyopathy in individuals harboring a deleterious genetic variant compared with non-carriers [7]. In this way, those patients who harbor a rare variant classified as P/LP in a DCM-related gene had an increased risk of suffering the most severe phenotypes [8]. Recently, Stroecks et al. suggested that a variant classified as ambiguous (variant unknown significance, VUS) in a robust DCM-associated gene is associated with an adverse prognosis, reinforcing the use of diagnostic gene panels only limited to definite DCM-associated genes [9]. These recent data highlight the crucial role of performing a genetic analysis with a suitable genetic interpretation. In addition, identification of a P/LP alteration allows familial screening to identify carriers, and consequently, those at risk of disease. Therefore, updating variants classified as not following ACMG recommendations is highly recommended [10]. In addition, a close periodic cardiac follow-up should be performed on relatives who carry the deleterious variant for early detection of the phenotype and the adoption of personalized measures [11].

Current guidelines recommend genetic diagnosis, even in the absence of familial context or associated clinical features [2,12,13]. However, it is not always conclusive, due to lack of variant identification in any of the currently known genes or due to the identification of a VUS following ACMG recommendations. The patient’s clinical history and family information are essential for the interpretation of variants but a lack of data or controversial available data impedes a conclusive classification [14]. Due to dynamic, continuous improvements in the data concerning rare variants, their reinterpretation may modify previous classifications, clarifying their clinical role, especially in rare variants previously classified as VUS. Thus, it may alter patient diagnosis and treatment, prenatal diagnosis or pre-implantation genetic diagnosis, and the screening of at-risk family members.

Up to now, few studies have focused on how often reanalysis is necessary and what the management implications are in inherited arrhythmogenic syndromes [15,16], as well as familial cardiomyopathies [17]. In DCM, few studies focused on variant reevaluation are published nowadays, showing nearly a 30% decrease in ambiguity in VUSs classified more than ten years ago [18]. However, a large burden of ambiguous variants remains. The development of improved tools to clarify the role of VUS is necessary [8,19]. In this approach, we focused on stringent gene selection for DCM genetic testing, helping to reduce the number of VUSs. We move one step forward and propose a new algorithm including definite-gene DCM, and also the parameters of population frequencies which aim to clarify this critical point, facilitating accurate genetic classification in G-DCM patients.

## 2. Results

Our retrospective study included 65 non-related Caucasian patients with a definitive clinical diagnosis of DCM. Patients were diagnosed between 2016 and 2019, according to the clinical guidelines available at the time of the study. Exhaustive genetic analysis of all genes related to DCM identified at least one rare variant classified as a VUS, following ACMG recommendations. We reinterpreted all rare variants according to the present available data.

**Figure 1:**
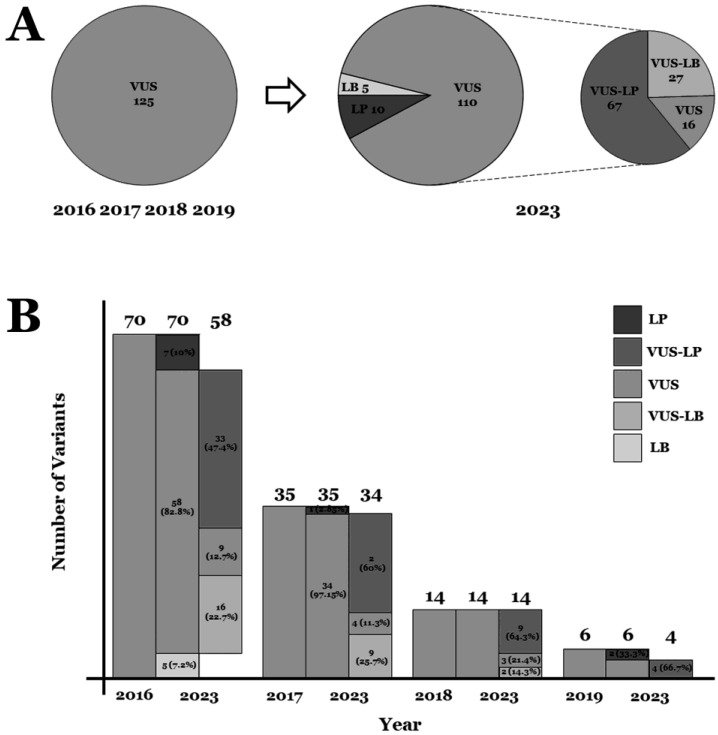
Global number/percentage of VUSs. (**A**) Global reclassification of variants. (**B**) Reclassification each year. LB: likely benign. VUS: variant of unknown significance. VUS-LP: variant of unknown significance—likely pathogenic. VUS-LB: variant of unknown significance—likely benign.

A total of 125 rare variants were located in 16 different genes with a definite association with DCM: one in *ACTC1* (0.8%), three in *ACTN2* (2.4%), one in *BAG3* (0.8%), one in *DES* (0.8%), four in *DSP* (3.2%), two in *FLNC* (1.6%), two in *JPH2* (1.6%), four in *LMNA* (3.2%), five in *MYH7* (4%), six in *RBM20* (4.8%), two in *SCN5A* (1.6%), two in *TNNC1* (1.6%), one in *TNNI3* (0.8%), six in *TNNT2* (4.8%), eighty-one in *TTN* (64.8%), and four in *VCL* (3.2%). Most of the rare variants were missense (95 variants, 76%), and eleven indels (8.8%) including one gross deletion, seven intronic variants (5.6%), and twelve nonsense variants (9.5%), including one stoploss (Table 1; Figure 1).

**Figure 2:**
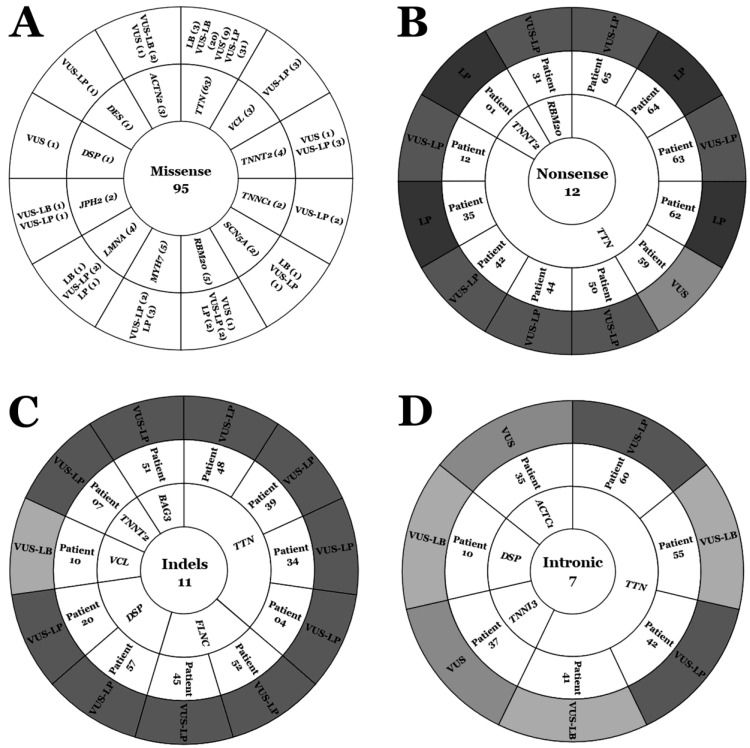
Number, type, and classification of variants. (**A**) Missense variants. (**B**) Nonsense variants. (**C**) Indel variants. (**D**) Intronic variants. LB: likely benign. VUS: variant of unknown significance. VUS-LP: variant of unknown significance—likely pathogenic. VUS-LB: variant of unknown significance—likely benign.

Our comprehensive analysis showed that 15 of the 125 rare variants (12%) were reclassified; five variants downgraded their role to LB and ten upgraded their level to LP. All rare variants downgraded the level of pathogenicity were missense (one in *LMNA*, one in *SCN5A*, and three in *TTN*) and modifications were due to increased MAF from the previous to current classification. Concerning the ten rare variants upgraded to LP, four were nonsense (one in *TNNT2*, three in *TTN*) and six missenses (one in *LMNA*, three in *MYH7*, two in *RBM20*). The 110 rare variants previously classified as VUS remain currently as VUS. At this point, we developed three subgroups within the VUS category, as mentioned in the Methods section. We observed that 27 (24.54%) variants were reclassified as VUS-LB and 67 (60.9%) as VUS-LP. The remaining 16 (14.54%) rare variants remain as VUSs (Table 1 and Table 2; Figure 1 and Figure 2).

We also performed an analysis by gene (*ACTC1*, *ACTN2*, *BAG3*, *DES*, *DSP*, *FLNC*, *JPH2*, *LMNA*, *MYH7*, *RBM20*, *SCN5A*, *TNNC1*, *TNNI3*, *TNNT2*, *TTN*, and *VCL*) and type of variant (missense, intronic, indels, and nonsense), as well as each year of previous classification (2016, 2017, 2018, and 2019) compared to current classification. Firstly, concerning years of previous classification, we observed that the further away that year was (2016 in our study), the more variability we found compared to 2023, after applying our algorithm. Additionally, the closer the year of classification to now, the greater the proportion of VUSs showed a tendency toward potential pathogenicity, as well as a lower tendency to play a potential benign role in DCM. Therefore, out of 70 VUS in 2016, five (7.14%) decreased to LB, and seven increased their pathogenicity to LP (10%). Inside the group of the remaining 58 VUSs, 33 seemed to show a tendency for increasing their potential deleterious role (56.89%) and 16 showed a tendency for decreasing their role to benignity (27.58%). In 2017, one of 35 increased its definitive role to LP (2.85). Of the remaining 34 VUSs, 21/34 seemed to show a tendency for the potential deleterious role (61.76%) and 9/34 a tendency for benignity (26.47%). In 2018, from the fourteen VUSs, nine seemed to show a tendency for a potential deleterious role (64.3%) and two a tendency for being not-deleterious (14.3%). In 2019, the last year included in our study, two VUSs showed a tendency toward LP (33.33%). The remaining four VUSs showed a tendency for a potential deleterious role (VUS-LP). Regarding the type of variant, most of those previously classified as VUSs that nowadays showed a tendency to increase their deleterious role to LP were missense (4.8%) and nonsense (3.2%). A group of sixty-seven variants (forty-eight missense, ten indels, seven nonsense and two intronic) showed a tendency to be deleterious (VUS-LP) after applying our algorithm (Table 1 and Table 2; Figure 1 and Figure 2).

Additionally, we performed an exhaustive analysis focused on *TTN*, the major gene currently associated with DCM. We identified that 3 of 81 rare variants changed from VUS to LB (3.7%). These three variants were missense and were previously classified in 2016. Three more rare variants upgraded to LP, which were all nonsense. After applying our algorithm to the remaining 75 VUSs, 22 (29.33%) showed a tendency to not be deleterious (VUS-LB), 20 of which were missense and 2 intronic, and 43 (57.33%) seemed to have a potential deleterious role despite not being confirmed (VUS-LP), of which thirty-one were missense, six nonsense, four indels, and two intronic. Concerning the proportion of potential pathogenicity, indels and nonsense showed high levels (100% and 90%, respectively), whereas missense and intronic showed medium rates (63.49% and 50%, respectively) (Table 1 and Table 2; Figure 1 and Figure 2).

Finally, we identified ten patients harboring an LP variant (six harboring only an LP variant and four more harboring one LP variant plus at least one VUS-LP). All these deleterious variants were located in *LMNA* (one variant), *MYH7* (three variants), *RBM20* (one variant), *TNNT2* (one variant), and *TTN* (six variants) genes. Our algorithm showed that 44 patients carried at least one rare variant classified as VUS-LP. A total of sixty-three variants with a highly potential deleterious role were identified (forty-four missense, eleven indels, seven nonsense, and one intronic). These rare variants with a potential deleterious role were located in the same genes abovementioned, as well as *BAG3*, *DES*, *DSP*, *FLNC*, *JPH2*, *SCN5A*, *TNNC1*, and *VCL* (Table 1 and Table 2; Figure 2).

## 3. Discussion

In the era of precision medicine, conclusive molecular genetic tests inform diagnoses, prognoses, and risk assessments for patients and their relatives in G-DCM [1]. The use of current ACMG recommendations helps to obtain a classification of rare variants [6]. However, an implicit stringent variant adjudication approach and a lack of sufficient scientific data, or available but controversial data, lead to many rare variants remaining categorized as VUSs. This ambiguous role does not provide conclusive data on the cause of disease and cannot be disregarded [5]. Continuous data improvement may modify a previous ambiguous classification, helping to gain certainty and, therefore, playing an actionable clinical role [2]. This fact highlights the importance of periodic reanalysis of VUS, at least in a timeframe from 2 to 5 years, as suggested by our group in previous studies [10,15,17,20]. Therefore, revising and clarifying the roles of VUSs is necessary for obtaining a comprehensive diagnosis [5]. In G-DCM, a sole study focused on variant reevaluation has been published to date, showing a near 30% decrease in ambiguity in VUS classified more than 10 years ago and without following ACMG recommendations [18]. A high percentage of reclassification suggests an immediate update of variants classified not following current ACMG recommendations, as already suggested by our group [10]. Another study showed a reduction in VUSs using analyses including only genes with a definitive-MCD association [19], demonstrating that the prior choice of genes to study is a key point, in our view, before performing a genetic analysis. Our study moved one step forward and designed an easy-to-use algorithm, helping to unravel the tendency of VUSs in G-DCM.

In our study, 12% of VUSs definitively modified their first classification, helping, thus, to clarify their role in G-DCM. This modification has been performed mainly by updating population frequencies; a simple and quick action. We suggest that it should be mandatory at least to update MAF periodically in public databases. We identified that 4% of VUSs decreased their ambiguous roles to benign. In concordance with previous studies, reducing the ambiguity of a VUS is an important advantage of a comprehensive reanalysis because these VUSs can be disregarded as the main cause of disease in each patient [17,20]. The 8% of VUSs increased the pathogenic potential to LP in G-DCM due to the reduction in MAF and, in a few cases, additional functional data since the first classification. All these variants are located in genes with a current definite/strong association with DCM [2]. However, no family segregation data were available in our study, impeding a conclusive translation into families. At this point, it is important to highlight the necessity of performing comprehensive large family correlation analysis to gain certainty. Another main item is functional studies. The use of this basic approach can help to unravel the pathophysiological mechanism of a rare variant, helping to conclude a definite pathogenic role. In recent years, the use of iPSC-derived cardiomyocytes, especially, has allowed researchers to demonstrate the molecular and cellular pathways involved in the pathogenicity of a rare variant. Both kinds of studies are crucial to help with clinical diagnosis, due to the fact that patients harboring a deleterious variant in any cardiomyopathy-associated gene showed a higher risk of mortality and a significantly increased risk of developing cardiomyopathy [7,8].

We identified that most of the rare variants remain classified as VUSs to date, following the current ACMG recommendations. This implies no impact on clinical management [1]. However, a recent study suggests that a VUS in a robust DCM-associated gene is associated with an adverse prognosis [21]. To shed light on the ambiguity implicit in VUSs, subgroups were categorized according to the abovementioned algorithm (please see methods section) already used in other inherited arrhythmogenic diseases [17,20], but now explicit for G-DCM, according to definite gene–disease association, lack of data or available contradictory data, and extremely rare population frequencies. Despite already being published, we suggest the additional use of our algorithm in other centers, and large cohorts in order to corroborate proper VUS subclassification. Hence, of the 110 rare variants that remain as VUSs after reclassification following the ACMG recommendations, 60.90% increase their potential pathogenic role (VUS-LP), 14.54% remain as VUSs, and 24.54% potentially decrease their ambiguous role (VUS-LB). It is important to remark that despite a tendency toward a benign or deleterious role in G-DCM, all these variants remain as VUSs and no clinical translation and adoption of therapeutic measures should be performed [1,2]. Unfortunately, for the VUS analyzed in our study, neither a complete family segregation nor a functional analysis is currently available, impeding a conclusive role following the current ACMG recommendations. It is important to mention a recent study suggesting that a VUS can be associated with an increased risk of cardiac mortality and heart failure hospitalization, especially those VUSs located in definite genes (*ACTC1*, *BAG3*, *DSP*, *FLNC*, *LMNA*, *MYH7*, *NEXN*, *PLN*, *RBM20*, *TNNC1*, *TNNT2*, *TPM1*, *TTN*, and *VCL*) [21]. In our study, 94.02% of reclassified VUS-LP variants were located in any of these genes with a robust association with G-DCM, supporting a potential deleterious role and suggesting a close follow-up of patients harboring any of these variants.

The reinterpreted variants were previously classified in different years, from 2016 to 2019, both inclusive. The percentage of reclassified variants was higher in 2016 than in 2019, in concordance with studies published several years ago in the field of reclassification of VUS associated with inherited arrhythmogenic syndromes [10,15,17,20]. This is mainly due to the constant updating of population frequencies, which allows us to have more precise data that brings us closer to being able to classify the variants more precisely, as occurred in previous studies [15,17,20]. Therefore, the number of variants remaining steady as the number of VUSs decreases constantly in comparison to previous years and, for this reason, the percentage of variants reclassified in 2019 was lower than in 2016, at least in our study, which is also in concordance with previous studies of our group in other inherited arrhythmogenic syndromes [15,17,20]. In this way, while being focused on updating population frequencies, the algorithm we propose also allows us to observe that VUSs with a tendency to be deleterious are increasingly classified today, while more variants with a tendency to have a genetic background without a main causative role can be ruled out. In G-DCM, we observed this pattern with a smaller number of VUSs each year, as we move closer to the present. In addition, and following specific algorithms for unravelling the role of VUSs in each disease, their classification is more precise, with a tendency to be deleterious, as reported in other inherited arrhythmogenic diseases [15,17,20]. It is important to mention that obtaining precise classifications occurs mainly due to updates in population frequencies, without including functional studies or genotype-phenotype correlations of large families for more than three generations. Despite these limitations, as already mentioned, the inclusion of these additional studies is necessary to be able to conclude a definitive pathogenic role in these rare variants in G-DCM [1,5].

Finally, it is important to remark that most of deleterious or potentially deleterious variants were identified in the *TTN* gene, the main gene associated with G-DCM in adult and pediatric population. Re-evaluation was also higher in *TNNT2*, *RBM20*, *MYH7*, and *LMNA*, in concordance with reports related to aggressive phenotypes, especially in the pediatric population [22,23]. Therefore, if a variant is re-analyzed in a pediatric patient, it should be also taken into account. Focused on *TTN*, the major gene currently associated with DCM [1,2], despite this ratio being based mainly on radical variants (nonsense, indels…) [24]. Most of the reported VUSs in the *TTN* gene are missense [25], as observed in our study. The role of *TTN* missense variants in G-DCM has been difficult to elucidate because of a lack of data concerning this gene, which encodes the largest protein in the human body and is associated with several skeletal muscular diseases [26]. This lack of data impedes a conclusive classification of most rare variants, clarifying a definite role of only three missense variants to LB and three nonsense variants to LP. After applying our specific algorithm for G-DCM, and updating only population frequencies, practically all nonsense and indel variants increased their potential deleterious role to VUS-LP in contrast to nearly 50% of intronic and missense VUSs. We moves one step forward in the prediction of missense VUS in *TTN*, contrary to a study suggesting that *TTN* missense variants should be classified as likely benign due to lack of data [27]. Our approach suggests a tendency for a potential deleterious role, but it is necessary to include functional and large family segregation studies to clarify the role of missense variants in *TTN*, according to a recent study [28]. For this reason, we suggest that the adoption of measures based on missense variants in *TTN* should be undertaken with caution and in a personalized way.

There are some limitations to mention. The reclassification and reinterpretation of rare genetic variants in our cohort have important limitations. Firstly, all cases included in our cohort may carry additional rare variants in other genes not included in our panel. However, we have analyzed all genes currently associated with G-DCM. In addition, a lack of available functional data for most rare variants impedes a more accurate interpretation. In this same way, a lack of family segregation, which is also crucial to clarify the role of a rare variant, in our view, is a key point to finally conclude the potential deleterious role of a rare variant. To support our results, reclassifications/reinterpretations of all variants should be corroborated in additional larger cohorts of G-DCM patients, to clarify their potential actionable role in clinical practice. At this point, it is important to take into account the ethnicity of the patient, due to genetic differences from diverse ancestries, to guide testing interpretation [29]. Finally, who should assume to perform the reclassification/reinterpretation and the implicit economic cost are controversial points not assessed in our study.

## 4. Materials and Methods

### 4.1. Study Cohort

Our retrospective study included 65 patients (all adults, >18 years old; mean age: 43.2 years old; 61.23% men; all Caucasian) with a definitive clinical diagnosis of DCM, determined following the available clinical guidelines [1,30,31]. Suspected cases with an inconclusive diagnosis were not included, to avoid bias in the reclassification of genetic variants.

All patients were genetically analyzed (please see Section 4.2). All patients included in our study carried at least one rare variant classified as a VUS, according to the ACMG recommendations [6].

### 4.2. Genetic Analysis

Genetic analysis was approved by the Ethics Committee of Hospital Josep Trueta (Girona, Catalonia, Spain), following the World Medical Association Declaration of Helsinki. Clinical and genetic data on all the patients were anonymized and kept confidential. Written informed consent was obtained from all the patients before genetic analysis.

A resequencing custom-made panel included all the main genes associated with G-DCM [2,5] (*ACTC1*, *ACTN2*, *BAG3*, *DES*, *DSP*, *FLNC*, *JPH2*, *LMNA*, *MYH7*, *NEXN*, *PLN*, *RBM20*, *SCN5A*, *TNNC1*, *TNNI3*, *TNNT2*, *TPM1*, *TTN*, and *VCL*). Isoforms of all the genes are described in Ensembl 75 (www.ensembl.org/), linked to a RefSeq code (www.ncbi.nlm.nih.gov/refseq/) or CCDS (www.ncbi.nlm.nih.gov/CCDS/). The bioinformatic analysis included adaptor and low-quality base trimming of the FASTQ files. GEM III was used for map-trimmed reads. The output was sorted, and uniquely and properly mapped read pairs were selected. SAMtools v.1.2 was used for variant calling from the cleaned BAM files. The final annotation steps provided information included in public databases. Rare genetic variants (with a minor allele frequency—MAF—of <1%) were confirmed by Sanger sequencing. The exons and exon–intron boundaries of each gene were amplified in both directions.

The SeqScape v2.7 software (Applied Biosystems, Waltham, MA, USA) was used to reanalyze all sequences obtained. No new rare variants were identified in any of the analyzed genes. The original classification compared rare variants with DNA sequences from 350 healthy Spanish individuals (individuals not related to any index case and of the same ethnicity) as control cases. Sequence variants were described following the Human Genome Variation Society (HGVS) rules (www.hgvs.org/). Currently, all rare variants in any of the genes analyzed were contrasted in the Genome Aggregation Database (gnomAD) (https://gnomad.broadinstitute.org/). All rare variants were also consulted in ClinVar (www.ncbi.nlm.nih.gov/clinvar/intro/), VarSome (www.varsome.com/), CardioClassifier (www.cardioclassifier.org/), InterVar (https://wintervar.wglab.org/), and CardioVAI (https://cardiovai.engenome.com/).

### 4.3. Data Sources

An exhaustive review of the available data on each rare variant was performed independently by five authors; subsequently, the reviews were compared and verified. Data were collected from the Human Gene Mutation Database (HGMD) (www.hgmd.org), ClinVar (https://www.ncbi.nlm.nih.gov/clinvar/intro/), the National Center for Biotechnology Information single-nucleotide polymorphism (SNP) database (https://www.ncbi.nlm.nih.gov/snp), Google Scholar (https://www.scholar.google.com), Springer Link (https://www.springer.com), and Science Direct (www.sciencedirect.com).

### 4.4. Classification/Interpretation

**Figure 3:**
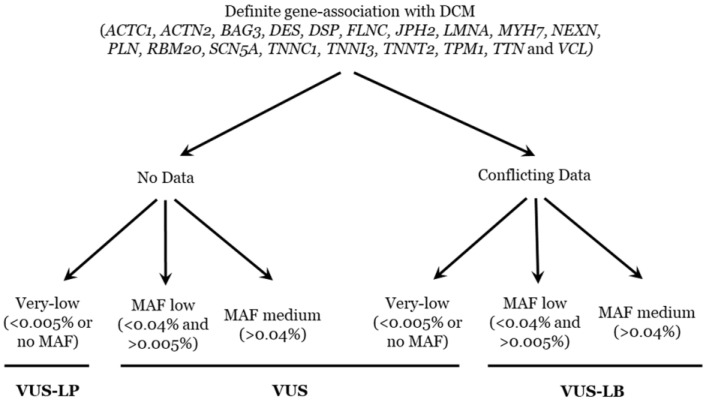
Algorithm of classification for VUS. MAF: minor allele frequency. VUS: variant of unknown significance. VUS-LP: variant of unknown significance—likely pathogenic. VUS-LB: variant of unknown significance—likely benign.

From 2016 to 2019, all rare variants in genes associated with DCM were classified according to the ACMG recommendations [6]. Now, the same rare variants have also been reclassified and the same ACMG recommendations also followed, but updates are included: the PM2 item was considered fulfilled if the MAF in the relevant population databases was ≤0.01% [32]. In addition, all reported deleterious variants currently classified as definitively pathogenic in IASs following ACMG recommendations are very rare (MAF < 0.005%) [33]. Other recent updates, according to gene-specific [34] and disease-specific associations, have also been taken into account [35,36]. The final classification of a rare variant as a VUS may be due to a lack of data or incongruences in the available data [37,38]. The current prevalence for familial DCM is approximately 1/2500 (MAF: 0.04%). Taking into account these two items, we propose a subclassification of VUSs into three subgroups: VUS-LB, VUS-LP, or remaining VUS (please see Figure 3). The first point is a definite gene-association with DCM (*ACTC1*, *ACTN2*, *BAG3*, *DES*, *DSP*, *FLNC*, *JPH2*, *LMNA*, *MYH7*, *NEXN*, *PLN*, *RBM20*, *SCN5A*, *TNNC1*, *TNNI3*, *TNNT2*, *TPM1*, *TTN*, and *VCL*) [2]. Then, the existence or not of data (if available, contradictory data). Finally, the use of the MAF (prevalence of familial DCM is the threshold), being very low (<0.005% or no MAF), low (>0.005% and <0.04%), or medium (>0.04%). We have already implemented this approach in previously published manuscripts focused on other IAS [16,20,21]. Genetic data were independently evaluated and classified by five authors, who are specialists in the genetics of inherited arrhythmias, to avoid bias. All investigators agreed on the final classification of all the rare variants included in our study.

## 5. Conclusions

In summary, a definite genetic diagnosis of rare variants related to G-DCM has actionable consequences for patients and their relatives. This emphasizes the importance of cautious genetic interpretation of variants and highlights the necessity of periodic reclassification/reinterpretation if a variant remains as a VUS, at least once every five years. Part of VUS clarifies its function after an update of population frequencies; a simple, cheap, and fast action. Due to a lack of functional studies as well as comprehensive clinical studies of large familial cases, most rare variants remain as VUSs in G-DCM following the stringent criteria suggested in ACMG recommendations. Identifying a deleterious or benevolent tendency of a VUS help with follow-up patients and their relatives despite no clinically actionable, so far. We propose a specific algorithm to help the interpretation of VUSs in G-DCM.

## Figures and Tables

**Table 1:** Genetic data of variants.

Patient	Gene	Nucleotide	Protein	dbSNP	ClinVar	GnomAD (MAF%)	Classification (Year)	Classification 2023
1	*TNNT2*	c.860G>A	p.Trp287Ter	rs727504247	LP	NA	VUS (2016)	LP
1	*TTN*	c.47951G>A	p.Arg15984His	rs201774108	VUSc	0.0001	VUS (2016)	VUS
2	*TTN*	c.73195G>A	p.Val24399Ile	rs1257567608	NA	NA	VUS (2016)	VUS (VUS-LP)
2	*TTN*	c.57388C>T	p.Arg19130Cys	rs72646861	LB	0.827	VUS (2016)	LB
2	*TTN*	c.53117C>T	p.Pro17706Leu	rs72646845	LB	0.369	VUS (2016)	LB
3	*ACTN2*	c.2051A>T	p.Asn684Ile	rs576783493	VUSc	0.0001	VUS (2016)	VUS
3	*TTN*	c.93125G>A	p.Gly31042Asp	rs373754986	VUS	0.007	VUS (2016)	VUS (VUS-LP)
3	*TTN*	c.76456G>C	p.Asp25486His	rs780958039	VUS	0.0008	VUS (2016)	VUS (VUS-LP)
4	*ACTN2*	c.1426G>A	p.Ala476Thr	rs142943120	VUSc	0.027	VUS (2016)	VUS (VUS-LB)
4	*TTN*	c.57978del	p.Val19327PhefsTer10	NA	NA	NA	VUS (2016)	VUS (VUS-LP)
4	*TTN*	c.76559G>A	p.Ser25520Asn	rs200450022	VUSc	0.061	VUS (2016)	VUS (VUS-LB)
4	*TTN*	c.72764G>C	p.Gly24225Ala	rs114071241	VUS	0.001	VUS (2016)	VUS (VUS-LP)
4	*TTN*	c.17066G>C	p.Gly5689Ala	rs200118743	VUSc	0.063	VUS (2016)	VUS (VUS-LB)
4	*TTN*	c.4675G>A	p.Val1559Ile	rs538451328	NA	0.0003	VUS (2016)	VUS (VUS-LP)
5	*TTN*	c.98971G>C	p.Glu32991Gln	rs199632397	VUSc	0.042	VUS (2016)	VUS (VUS-LB)
5	*TTN*	c.27659G>A	p.Arg9220Gln	rs727504757	VUS	0.003	VUS (2016)	VUS (VUS-LP)
6	*TTN*	c.45392G>A	p.Arg15131His	rs72646808	VUSc	0.185	VUS (2016)	LB
7	*TNNT2*	c.629_631del	p.Lys210del	rs45578238	VUS	NA	VUS (2016)	VUS (VUS-LP)
8	*MYH7*	c.1106G>A	p.Arg369Gln	rs397516089	LP	0.00006	VUS (2016)	LP
9	*TTN*	c.73967A>G	p.Asn24656Ser	rs368443217	VUSc	0.008	VUS (2016)	VUS (VUS-LB)
9	*TTN*	c.65012T>A	p.Met21671Lys	rs750298083	VUSc	0.004	VUS (2016)	VUS
9	*TTN*	c.51830G>A	p.Arg17277His	rs201457934	VUSc	0.01	VUS (2016)	VUS (VUS-LB)
9	*TTN*	c.9247G>A	p.Glu3083Lys	NA	NA	NA	VUS (2016)	VUS (VUS-LP)
9	*MYH7*	c.2711G>A	p.Arg904His	rs397516165	LP	0.0001	VUS (2016)	LP
10	*DSP*	c.1266+6G>T	NA	rs73375345	LB	0.037	VUS (2016)	VUS (VUS-LB)
10	*TTN*	c.20920A>G	p.Ser6974Gly	rs72648980	VUSc	0.062	VUS (2016)	VUS (VUS-LB)
10	*VCL*	c.2862_2864del	p.Leu955del	rs397517237	VUSc	0.021	VUS (2016)	VUS (VUS-LB)
11	*SCN5A*	c.6010T>C	p.Phe2004Leu	rs41311117	VUSc	0.198	VUS (2016)	LB
12	*TTN*	c.72970C>T	p.Gln24324Ter	NA	NA	NA	VUS (2016)	VUS (VUS-LP)
13	*TTN*	c.49249G>A	p.Asp16417Asn	rs1244503464	NA	0.0004	VUS (2016)	VUS (VUS-LP)
14	*TTN*	c.12814G>T	p.Asp4272Tyr	rs72648940	VUSc	0.0005	VUS (2016)	VUS
15	*TTN*	c.77076A>C	p.Glu25692Asp	rs370547473	NA	0.004	VUS (2016)	VUS (VUS-LP)
16	*TTN*	c.32932C>A	p.Pro10978Thr	rs1393076582	VUSc	NA	VUS (2016)	VUS
17	*DES*	c.179C>T	p.Ser60Leu	rs868853251	NA	0.0001	VUS (2016)	VUS (VUS-LP)
17	*TTN*	c.46877G>A	p.Gly15626Asp	rs201802447	VUS	0.007	VUS (2016)	VUS
17	*TTN*	c.1066G>C	p.Glu356Gln	rs144531477	VUSc	0.015	VUS (2016)	VUS (VUS-LB)
18	*TTN*	c.95137A>G	p.Ile31713Val	rs758945559	NA	0.0008	VUS (2016)	VUS (VUS-LP)
18	*TTN*	c.30484C>A	p.Pro10162Thr	rs532102837	VUSc	0.058	VUS (2016)	VUS (VUS-LB)
18	*DSP*	c.6799A>T	p.Thr2267Ser	rs181378432	LB	0.009	VUS (2016)	VUS
18	*LMNA*	c.1621C>T	p.Arg541Cys	rs56984562	LP	0.0001	VUS (2016)	LP
19	*RBM20*	c.1900C>T	p.Arg634Trp	rs796734066	LP	NA	VUS (2016)	LP
20	*DSP*	del ex. 21_24	NA	NA	NA	NA	VUS (2016)	VUS (VUS-LP)
21	*MYH7*	c.1371A>G	p.Ile457Met	NA	NA	NA	VUS (2016)	VUS (VUS-LP)
22	*TTN*	c.94724T>C	p.Met31575Thr	rs397517786	VUSc	0.007	VUS (2016)	VUS (VUS-LB)
22	*TTN*	c.62687G>T	p.Gly20896Val	rs549938348	VUS	0.0004	VUS (2016)	VUS (VUS-LP)
23	*RBM20*	c.1906C>T	p.Arg636Cys	rs267607002	VUSc	0.0001	VUS (2016)	VUS
24	*JPH2*	c.1736C>T	p.Pro579Leu	rs953353202	VUS	0.001	VUS (2016)	VUS (VUS-LP)
24	*RBM20*	c.1980C>A	p.Ser660Arg	NA	NA	0.00007	VUS (2016)	VUS (VUS-LP)
25	*RBM20*	c.2200C>G	p.Arg734Gly	NA	NA	NA	VUS (2016)	VUS (VUS-LP)
26	*TTN*	c.57212G>A	p.Arg19071Gln	rs373282633	VUS	0.003	VUS (2016)	VUS
26	*TTN*	c.48221T>A	p.Leu16074Gln	rs140714512	VUSc	0.051	VUS (2016)	VUS (VUS-LB)
26	*TTN*	c.29645A>C	p.Lys9882Thr	rs760742068	VUS	0.001	VUS (2016)	VUS (VUS-LP)
27	*LMNA*	c.1930C>T	p.Arg644Cys	rs1420000963	VUSc	0.201	VUS (2016)	LB
27	*TTN*	c.77188C>T	p.Arg25730Trp	rs779581886	VUS	0.002	VUS (2016)	VUS (VUS-LP)
27	*TTN*	c.60581T>C	p.Leu20194Pro	rs1359881893	VUS	0.0008	VUS (2016)	VUS (VUS-LP)
28	*RBM20*	c.1913C>T	p.Pro638Leu	rs267607003	LP	0.0003	VUS (2016)	LP
28	*TTN*	c.74942C>T	p.Ala24981Val	rs749950083	NA	NA	VUS (2016)	VUS (VUS-LP)
28	*TTN*	c.73501C>G	p.Pro24501Ala	rs770542451	NA	0.0004	VUS (2016)	VUS (VUS-LP)
28	*TTN*	c.1333G>A	p.Ala445Thr	rs142414432	VUSc	0.021	VUS (2016)	VUS (VUS-LB)
29	*LMNA*	c.1949A>G	p.Asn650Ser	rs775728847	VUS	0.0003	VUS (2016)	VUS (VUS-LP)
29	*TTN*	c.38807A>G	p.Asn12936Ser	rs1184631064	VUS	0.0008	VUS (2016)	VUS (VUS-LP)
30	*ACTN2*	c.1040C>T	p.Thr347Met	rs727504590	VUSc	0.009	VUS (2016)	VUS (VUS-LB)
31	*RBM20*	c.3684A>G	p.Ter1228TrpextTer33	rs1845123103	NA	0.0001	VUS (2016)	VUS (VUS-LP)
31	*TTN*	c.40001G>A	p.Gly13334Glu	rs561284948	VUS	0.001	VUS (2016)	VUS (VUS-LP)
31	*TTN*	c.20863C>T	p.Pro6955Ser	rs1438804317	NA	NA	VUS (2016)	VUS (VUS-LP)
32	*MYH7*	c.602T>C	p.Ile201Thr	rs397516258	LP	0.0001	VUS (2016)	LP
32	*TTN*	c.71188G>A	p.Gly23730Arg	rs72648205	VUSc	0.034	VUS (2016)	VUS (VUS-LB)
33	*TTN*	c.82400G>A	p.Arg27467His	rs199895320	VUS	0.002	VUS (2016)	VUS (VUS-LP)
33	*TTN*	c.75575A>T	p.Asn25192IIe	rs200714263	VUS	0.002	VUS (2016)	VUS (VUS-LP)
34	*TTN*	c.9139_9150del	p.Ser3047_Thr3050del	NA	NA	NA	VUS (2016)	VUS (VUS-LP)
35	*ACTC1*	c.455-7C>T	NA	rs768363857	LB	0.003	VUS (2017)	VUS
35	*TTN*	c.53791C>T	p.Arg17931Ter	rs869312112	LP	NA	VUS (2017)	LP
35	*TTN*	c.33568T>G	p.Cys11190Gly	NA	NA	NA	VUS (2017)	VUS (VUS-LP)
36	*TTN*	c.58846G>A	p.Gly19616Ser	rs1262240030	NA	NA	VUS (2017)	VUS (VUS-LP)
36	*TTN*	c.58590G>C	p.Gln19530His	NA	NA	NA	VUS (2017)	VUS (VUS-LP)
36	*TTN*	c.8320G>A	p.Glu2774Lys	rs763666119	LB	0.0003	VUS (2017)	VUS (VUS-LP)
37	*TNNI3*	c.-8G>A	NA	rs773513015	VUSc	0.001	VUS (2017)	VUS
37	*TTN*	c.40796G>A	p.Arg13599Gln	rs778774812	VUS	0.001	VUS (2017)	VUS (VUS-LP)
38	*TTN*	c.57709G>T	p.Val19237Leu	rs1397460981	NA	NA	VUS (2017)	VUS (VUS-LP)
39	*TTN*	c.95967dup	p.Arg31990ThrfsTer10	NA	NA	NA	VUS (2017)	VUS (VUS-LP)
40	*TTN*	c.12389G>A	p.Cys4130Tyr	rs375577529	VUSc	0.008	VUS (2017)	VUS (VUS-LB)
40	*VCL*	c.2905G>A	p.Ala969Thr	rs199751261	VUS	0.002	VUS (2017)	VUS (VUS-LP)
41	*LMNA*	c.253C>G	p.Leu85Val	NA	NA	NA	VUS (2017)	VUS (VUS-LP)
41	*TTN*	c.78602G>A	p.Gly26201Asp	rs756222422	NA	0.0004	VUS (2017)	VUS (VUS-LP)
41	*TTN*	c.64137G>C	p.Lys21379Asn	rs56019808	VUSc	0.014	VUS (2017)	VUS (VUS-LB)
41	*TTN*	c.36844+9A>G	NA	rs372725070	LB	0.013	VUS (2017)	VUS (VUS-LB)
41	*TTN*	c.21355G>T	p.Ala7119Ser	rs200972189	VUSc	0.02	VUS (2017)	VUS (VUS-LB)
42	*TTN*	c.92472G>C	p.Lys30824Asn	NA	NA	NA	VUS (2017)	VUS (VUS-LP)
42	*TTN*	c.25810C>T	p.Arg8604Ter	NA	NA	NA	VUS (2017)	VUS (VUS-LP)
42	*TTN*	c.28738+7T>G	NA	NA	NA	NA	VUS (2017)	VUS (VUS-LP)
42	*TTN*	c.14656A>T	p.Thr4886Ser	rs794727816	VUS	NA	VUS (2017)	VUS (VUS-LP)
43	*TNNC1*	c.400G>A	p.Glu134Lys	rs1553651640	VUS	NA	VUS (2017)	VUS (VUS-LP)
43	*TTN*	c.95173A>G	p.Lys31725Glu	rs72629783	VUSc	0.021	VUS (2017)	VUS (VUS-LB)
43	*TTN*	c.31709T>C	p.Ile10570Thr	rs72650057	VUSc	0.013	VUS (2017)	VUS (VUS-LB)
44	*TTN*	c.56541G>A	p.Trp18847Ter	NA	LP	NA	VUS (2017)	VUS (VUS-LP)
45	*FLNC*	c.3612del	p.His1205ThrfsTer65	NA	NA	NA	VUS (2017)	VUS (VUS-LP)
46	*TTN*	c.19570G>A	p.Asp6524Asn	rs72648973	VUSc	0.075	VUS (2017)	VUS (VUS-LB)
47	*TNNT2*	c.476G>A	p.Arg159Gln	rs45501500	VUSc	NA	VUS (2017)	VUS
48	*TTN*	c.51369_51384del	p.Asp17123GlufsTer4	NA	NA	NA	VUS (2017)	VUS (VUS-LP)
49	*JPH2*	c.572C>G	p.Pro191Arg	rs554853074	LB	0.045	VUS (2017)	VUS (VUS-LB)
49	*TNNT2*	c.230C>T	p.Pro77Leu	rs144900708	VUS	0.003	VUS (2017)	VUS (VUS-LP)
50	*TTN*	c.86270C>A	p.Ser28757Ter	NA	LP	NA	VUS (2017)	VUS (VUS-LP)
50	*TTN*	c.62666A>T	p.Asp20889Val	rs535816123	NA	0.002	VUS (2017)	VUS (VUS-LP)
50	*TTN*	c.47435T>C	p.Ile15812Thr	rs72646819	VUSc	0.007	VUS (2017)	VUS (VUS-LB)
50	*TTN*	c.16066A>G	p.Thr5356Ala	rs530353051	VUS	0.002	VUS (2017)	VUS
51	*BAG3*	c.903del	p.Arg301SerfsTer6	NA	NA	NA	VUS (2018)	VUS (VUS-LP)
51	*TTN*	c.29327A>G	p.Tyr9776Cys	rs72650035	VUSc	0.02	VUS (2018)	VUS (VUS-LB)
52	*FLNC*	c.1414del	p.Cys472ValfsTer20	NA	NA	NA	VUS (2018)	VUS (VUS-LP)
53	*MYH7*	c.5452C>T	p.Arg1818Trp	rs763073072	VUS	0.0005	VUS (2018)	VUS (VUS-LP)
54	*TNNC1*	c.394G>A	p.Asp132Asn	rs397516846	VUS	0.0003	VUS (2018)	VUS (VUS-LP)
55	*TTN*	c.24617A>G	p.Asn8206Ser	NA	NA	NA	VUS (2018)	VUS
55	*TTN*	c.12889+7A>T	NA	rs10200398	VUSc	0.07	VUS (2018)	VUS (VUS-LB)
56	*TTN*	c.20839G>A	p.Glu6947Lys	rs201326258	VUS	0.003	VUS (2018)	VUS
56	*VCL*	c.1620T>G	p.Asp540Glu	rs533622785	NA	0.0006	VUS (2018)	VUS (VUS-LP)
57	*DSP*	c.5673_5674dup	p.Lys1892ArgfsTer38	NA	NA	NA	VUS (2018)	VUS (VUS-LP)
57	*SCN5A*	c.2924G>A	p.Arg975Gln	rs753149586	VUS	0.005	VUS (2018)	VUS (VUS-LP)
58	*TNNT2*	c.835G>A	p.Gly279Arg	rs757664792	VUS	0.0004	VUS (2018)	VUS (VUS-LP)
59	*TTN*	c.9220C>T	p.Arg3074Ter	rs780706937	VUSc	NA	VUS (2018)	VUS
60	*TTN*	c.81493+1G>T	NA	NA	NA	NA	VUS (2018)	VUS (VUS-LP)
61	*TNNT2*	c.391C>G	p.Arg131Gly	rs74315380	LP	NA	VUS (2019)	VUS (VUS-LP)
62	*TTN*	c.78412C>T	p.Arg26138Ter	rs794729384	LP	0.0004	VUS (2019)	LP
63	*TTN*	c.67528G>T	p.Glu22510Ter	NA	NA	NA	VUS (2019)	VUS (VUS-LP)
64	*TTN*	c.39790C>T	p.Arg13264Ter	rs751746401	LP	0.0004	VUS (2019)	LP
65	*TTN*	c.59787G>A	p.Trp19929Ter	NA	NA	NA	VUS (2019)	VUS (VUS-LP)
65	*VCL*	c.158A>G	p.Asn53Ser	rs751938777	VUS	0.001	VUS (2019)	VUS (VUS-LP)

Genetic data of variants. LB: Likely Benign. LP: Likely Pathogenic. MAF: Minor Allele Frequency. NA: Not Available. VUS: Variant of Unknown Significance. VUSc: Variant of unknown significance with Controversial/Conflicting data. VUS-LP: Variant of Unknown Significance-Likely Pathogenic. VUS-LB: Variant of Unknown Significance-Likely Benign.

**Table 2:** Type, and classification of genetic variants.

2023		Intronic	Indels	Nonsense	Missense	Total	
**B**		0	0	0	0	0	
**LB**		0	0	0	5 (4%)	5 (4%)	
**VUS**	**VUS-LB**	3 (2.4%)	1 (0.8%)	0	23 (18.4%)	27 (21.6%)	110 (88%)
	**VUS**	2 (1.6%)	0	1 (0.8%)	13 (10.4%)	16 (12.8%)	
	**VUS-LP**	2 (1.6%)	10 (8%)	7 (5.6%)	48 (38.4%)	67 (53.6%)	
**LP**		0	0	4 (3.2%)	6 (4.8%)	10 (8%)	
**P**		0	0	0	0	0	
**Total**		7 (5.6%)	11 (8.8%)	12 (9.6%)	95 (76%)	125 (100%)	

Type and classification of genetic variants. LB: likely benign. LP: likely pathogenic. VUS: variant of unknown significance. VUS-LP: variant of unknown significance—likely pathogenic. VUS-LB: variant of unknown significance—likely benign.

## Data Availability

All data are included in the manuscript.
